# Implementing a screening protocol for food insecure patients within a long‐term acute care hospital (LTACH): A community health needs assessment (CHNA)

**DOI:** 10.1002/ncp.11292

**Published:** 2025-04-16

**Authors:** Molly MacDonald, Christopher Stimson, Marti Samsel, Tina Gross

**Affiliations:** ^1^ Nutritional Sciences Department University of Michigan School of Public Health Ann Arbor Michigan USA; ^2^ University of Michigan Health‐Sparrow Specialty Hospital University of Michigan Health System Lansing Michigan USA

**Keywords:** adult, food insecurity, long‐term care, nutrition assessment, outcomes, public health, quality improvement, risk factors, social determinants of health

## Abstract

Food insecurity is a prevalent yet overlooked issue within the inpatient setting. Despite food insecurity being predictive of poor health outcomes and increased hospital readmission rates, the development of efficient screening protocols remains a gap in care for high‐risk, hospitalized patients. As part of a community health needs assessment (CHNA), a screening protocol for food insecurity has been implemented within a long‐term acute care hospital (LTACH). This is a multistep protocol in which each newly admitted patient is asked pertinent questions related to food insecurity during the initial nutrition consult, led by a registered dietitian, followed by dissemination of a nutrition‐resource packet upon positive screening. The packet includes a registry of county‐based community nutrition resources and samples of recommended oral nutrition supplements. Additionally, the interdisciplinary team is informed of positive food insecurity status during weekly rounding to facilitate appropriate coordination of care in preparation for patient discharge. During the first year of implementation, 2022–2023, an average 95% of identified food insecure LTACH patients per the CHNA screening protocol were provided the appropriate nutrition‐related resources during admission. This was improved upon from 2023‐2024 as 100% of LTACH patients identified as food insecure received appropriate CHNA nutrition resources. Further, 93% of food insecure patients captured from 2023–2024 reported living within geographical proximity, or within the same county as this healthcare facility. As this protocol continues to be refined, future considerations include embedding a brief, validated food insecurity questionnaire within the initial nutrition consult as well as tracking metrics related to protocol effectiveness.

## INTRODUCTION

Food insecurity is a pervasive yet elusive issue as it often goes unnoticed in the hospital setting. It is defined, per the Food and Agriculture Organization, as the inability to regularly access safe and nutritious food for normal development or lead an active and healthy lifestyle.^1^ Additionally, food insecurity is a known social determinant of health (SDoH) or a nonmedical, socially‐driven factor that can influence health outcomes.^2^ It has been shown in previous studies that patients who present to the hospital with food insecurity have an associated increase in emergency room (ER) visits, hospital readmissions, and longer length of stay (LOS) compared with food secure patient populations.^3^, ^4^, ^5^ Moreover, food insecure patients are less likely to see a primary care physician yet are twice as likely to utilize acute medical care within 90 days of hospital discharge compared with those who are not food insecure, making it a strong predictor of healthcare utilization.^6^ For example, food insecure patients incur an additional 11% in healthcare costs or nearly $2000 dollars more per person annually compared with food secure counterparts.^4^, ^5^, ^7^ Conversely, it has been demonstrated in a large, multisite, nutrition‐focused quality improvement project (QIP) that the provision of oral nutrition supplements (ONS) to nutritionally at‐risk, malnourished patients within home health agencies (*n* = 1546) led to a reduced relative risk of hospitalization within 30 (24.3%), 60 (22.8%), and 90 (18.3%) days post‐QIP enrollment as compared with historic controls (*n* = 7413).^8^ This is further supported by previous studies demonstrating that prioritizing ONS access to nutritionally at‐risk patients is effective in reducing unplanned hospitalization within 30 days of hospital discharge.^9^, ^10^

Despite the known health risks and financial burdens associated with food insecurity,^11^ implementing effective and standardized screening protocols during the hospital admission remains a gap in care. For instance, a 2019 national cross‐sectional study found that 39.8% of United States hospitals reported screening for food insecurity and only 24.4% of hospitals reported comprehensively screening for all SDoH domains (interpersonal violence, transportation needs, housing instability, etc).^12^ The events that occurred in response to the coronavirus 2019 pandemic, such as rising unemployment rates and increased food costs, only exacerbated rates of food insecurity while simultaneously placing strain on both the time and resources of healthcare personnel to conduct these screenings. For instance, the Economic Research Service within the US Department of Agriculture (USDA) reported that food insecure households with children increased from 13.6% to 14.8% within just 1 year, or from 2019 to 2020, which coincided with a marked increase in enrollment for federal food assistance programs^13^, ^14^ and these rates continue to rise present day.^15^ In response to this, improving food accessibility and reducing food insecurity serve as a central focus for nutrition and health‐based policies set by the White House National Strategy on Hunger, Nutrition, and Health. Initiatives include bolstering funding for research, expanding access to federal nutrition assistance programs, and pushing for universal screening of food insecurity within healthcare settings.^16^ However, while it is acknowledged that more robust screening methods are needed, reported barriers that exist within the hospital setting include time constraints, concern for stigmatization or marginalization of the patient, and the need for further resources and funding.^6^, ^17^

Low‐income patients who present to a long‐term acute care hospital (LTACH) are at particularly increased nutrition risk and can largely benefit from both food insecurity screening and nutrition‐focused resources. The LTACH patient population is characterized as being high acuity and requiring a complex level of medical care in the presence of wounds, infections, and multisystem failure.^18^ LTACH admission criteria includes the need for ventilatory support, medical management of severe wounds, or the presence of two condition‐specific indications such as dialysis secondary to end stage renal failure and/or intravenous antibiotic therapy due to infection.^18^, ^19^ The average LOS for the LTACH patient is at least 25 days, but there are patients who require extended periods of high‐level care and subsequently surpass this time frame.^19^ Optimizing their nutritional status throughout the entirety of the admission as well as continuing the prescribed medical nutrition therapy even after discharge is crucial for maximizing health outcomes. This identified nutrition‐centered gap in care has led to the implementation of a food insecurity‐focused community health needs assessment (CHNA) protocol which places community nutrition resources at the forefront of the nutrition care plan for all patients who present to this LTACH with food insecurity.

The objective of the CHNA protocol within this LTACH is to proactively implement a food insecurity screening process as part of the initial nutrition care plan and connect identified food insecure patients with appropriate nutrition‐related community resources during their hospital admission.

## MATERIALS AND METHODS

### Eligibility

This project was undertaken as a QIP at this institution and has been deemed exempt by our institutional review board (IRB# 2429). This is a 30‐bed LTACH located within a level 1 trauma center in a Midwest, urban city (Lansing, MI) that averages at least 400 patient admissions per year. Admission is reserved for patients who require high acuity medical care yet are anticipated to transition to a lower‐level of care within an averaged 25‐day period. Per LTACH protocol, every newly admitted patient must have an initial consultation completed by a registered dietitian (RD) within 72 hours of admission. The initial nutrition consultation comprehensively assesses the patients' nutritional status in the context of disease, wounds, infection, pre‐existing malnutrition status, and other underlying medical conditions, all of which drives the nutrition care plan throughout their admission.

The CHNA screening protocol adds another dimension to the initial nutrition consultation by adding a screening mechanism that considers whether the patient presents to the hospital with pre‐existing food insecurity. This preliminary screening protocol entails basic interview questions, which include, “Have you been struggling to access food prior to your admission?” or “Have you experienced financial concerns in regard to buying or obtaining food prior to this admission?” and “Do you utilize federally funded food programs (e.g. Meals on Wheels, Supplemental Nutrition Assistance Program [SNAP] or Women, Infants and Children, also known as WIC)?” If the patient responds positively to any of these questions, it signals as a qualifier for food insecurity.

Alternatively, there are cases in which patients will explicitly express, outside of the screening process, experiencing difficulty in affording and obtaining food on a regular basis. This would also serve as a qualifier or a positive screen for food insecurity. Further, some patients are admitted to the hospital directly from local homeless shelters and are well‐known to LTACH social workers as needing community resources. This would undoubtedly meet criteria for food insecurity, as well. There are also times in which the conversation surrounding food insecurity has occurred organically out of frustration from the patient when they are suggested to incorporate high‐quality protein sources in their diet or continue oral nutrition supplements after discharge. Food insecure patients will often express concern because they foresee that continuing the recommended medical nutrition therapy is not feasible outside of the hospital setting.

Ultimately, positive screening for food insecurity triggers (1) the dissemination of county‐specific CHNA nutrition resources and (2) RD‐led communication with the interdisciplinary team (e.g. physician and social workers) regarding food accessibility concerns.

### Nutrition‐related resources

All patients who meet criteria for food insecurity upon the initial nutrition assessment are offered a county‐specific CHNA nutrition‐related resource packet. Based on patientreported county of residence, a registry of local food pantries, senior centers, farmer's markets, and contact information for federal assistance programs (e.g. Double Up Food Bucks, SNAP benefits, WIC, Meals on Wheels) is provided. Social workers are additionally notified by the LTACH RD as to the patients who are provided this packet in case further assistance or guidance with program registration is needed during the hospital admission.

Additional components offered as part of the CHNA nutrition packet includes samples of scientifically and commercially available ONS as well as coupons for these products in an effort to help defray the cost of recommended nutrition‐related resources once the patient is discharged. The coupons provided are accepted at large retailers and the samples of protein supplements provided are based on RD recommendations (e.g. glucose‐control and/or high protein formulated products).

### Coordination of findings with the clinical team

A key aspect of the CHNA screening protocol includes the communication of positive food insecurity findings by the RD to the interdisciplinary team. This step in the protocol has been integrated into the weekly interdisciplinary team meetings which is a designated time in which the medical team systematically reviews each patient case. The interdisciplinary team foundationally includes LTACH RDs, social workers, physical therapists and occupational therapists, wound team nurses, nurse manager, nurse practitioner, physician (medical director), and the hospital chief executive officer. Relaying findings related to food insecurity with the entire medical team forms a more holistic plan of care as well as optimizes the coordination of prompt referrals, particularly as the patient approaches their discharge date or anticipates moving onto the next level of care.

## RESULTS

Prior to January 2022, there existed no standardized approach for food insecurity screening within this LTACH. This identified need ultimately led to the initiation and implementation of a food insecurity screening protocol as well as the dissemination of a county‐based nutrition‐resource packet to food insecure patients. Basic, observational information such as the patient's date of admission and the date that the CHNA nutrition‐related resources were provided was collected starting the first quarter of January 2022. Quarterly updates regarding the number of identified food insecure patients admitted within the hospital as well the proportion of admitted patients who successfully received these resources prior to discharge was reported by the LTACH RD to hospital administrative staff at scheduled quality improvement (QI) meetings.

**Figure 1:**
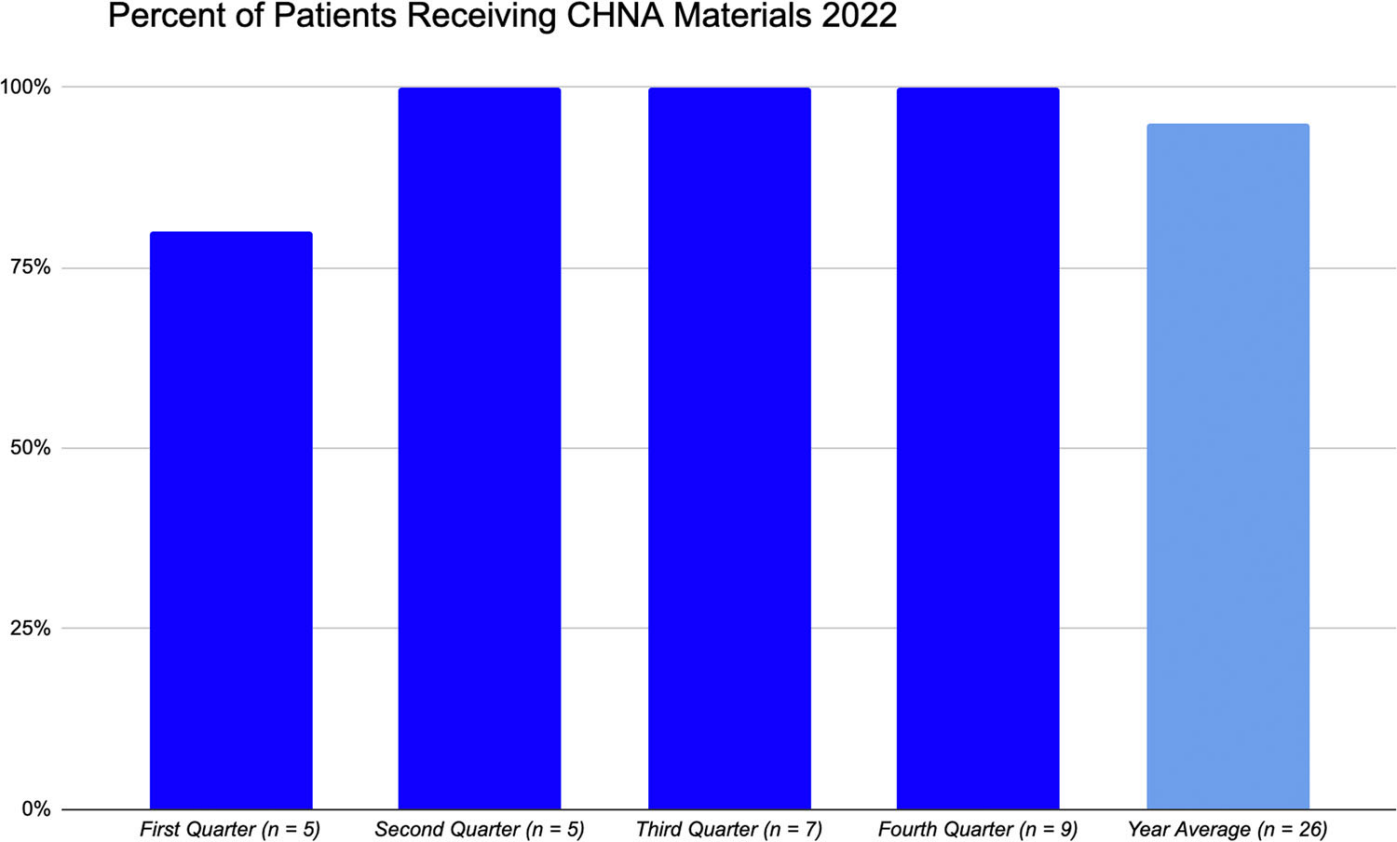
Percentage of identified food insecure long‐term acute care hospital (LTACH) patients receiving community health needs assessment (CHNA). Nutrition‐related materials by quarter and yearly average in 2022.

Within the first quarter of 2022, 80% of identified food insecure LTACH patients were provided pertinent nutrition‐related resources during their admission (five patients met criteria for food insecurity and one patient had been discharged to a rehabilitation facility before the RD was able to provide resources). For the remaining second through fourth quarters of 2022, every patient who screened positive for food insecurity was successfully captured and given the appropriate nutrition‐related resources before discharge. Therefore, for 2022, the year‐end quarterly average of patients provided CHNA‐related resources upon positive screening for food insecurity was 95% (*n* = 26) (see Figure 1 for details). From 2022 to 2023, this LTACH admitted a total of 425 patients, which indicates that approximately 6.1% of this patient population was identified as food insecure over the course of the year.

**Figure 2:**
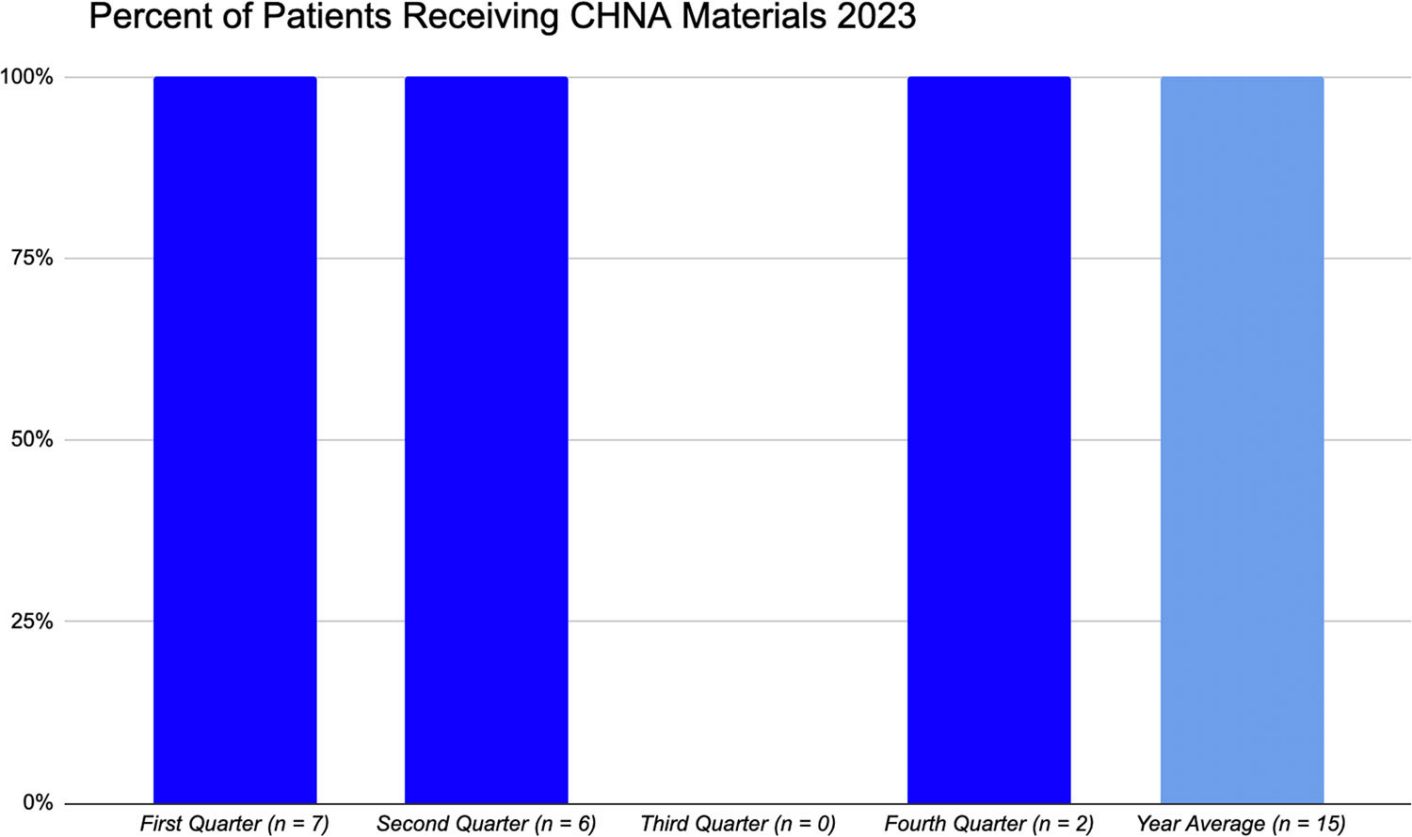
Percentage of identified food insecure long‐term acute care hospital (LTACH) patients receiving community health needs assessment (CHNA). Nutrition‐related materials by quarter and yearly average in 2023.

The following year, from 2023 to 2024, given that the protocol became a more standardized fixture within the initial RD consult, 100% of the identified patients were provided with the appropriate resources related to food insecurity before discharge (*n* = 15) (see Figure 2 for details). There were a total of 416 patient admissions within this LTACH from 2023 to 2024, indicating that 3.6% of the LTACH patient population were identified as food insecure during this time frame.

Interestingly, in evaluating patient‐reported counties of residence, it was found that the majority of the patients admitted to this LTACH and identified as food insecure from 2023 to 2024 resided within the same county as this facility. For instance, 14 out of the 15 (93%) of food insecure patients from 2023‐2024 reported living in Ingham County, MI. Of note, approximately 16.4% of the population within Ingham County live below the poverty line (comparatively, the national average is 12.5%)^20^ and the unemployment rate is 4.7%, which is slightly higher than the state average (4.5%) per the US Bureau of Labor Statistics.^21^, ^22^ Of note, before the pandemic, the Greater Lansing Food Bank had reported food insecurity rates of 13.8%, which surged to 19% by 2021.^23^ This speaks to the need for nutrition‐centered support within this community and thus the need for ongoing screening in community hospital settings.

## DISCUSSION

In the face of financial distress, food is often the first expense to be cut within a household or by an individual.^24^, ^25^ This lends to a cyclical deprioritization of high‐quality, nutrient‐dense foods (e.g. fruits, vegetables, whole grains), which tend to be replaced with highly processed foods that are void of nutrients and are obesogenic in nature.^26^, ^27^, ^28^ Chronic intake of nutrient‐poor, low‐quality foods can have deleterious health outcomes and exacerbate already existing clinical conditions such as hypertension, heart failure, wounds, infection, and malnutrition, all of which are common diagnoses encountered within the hospital setting.^29^, ^30^, ^31^, ^32^, ^33^, ^34^, ^35^ The LTACH patient population characteristically presents with a myriad of comorbid conditions that, compounded with their prolonged LOS, only place these patients at further nutrition risk.

Given that this is a known SDoH that can predict the likelihood of healthcare utilization and hospitalization rates,^6^ implementing a food insecurity screening protocol provides both clinical and economic value. This has been demonstrated by previous work done by Sulo et al. in which a budget impact model evaluating the cost savings of nutrition‐focused QIPs that were effective in reducing 30‐day hospital readmission rates and LOS led to a per‐patient net savings of nearly $1500 as compared to the baseline, pre‐QIP cohort.^36^ Broadly speaking, investing the time to address food insecurity in the inpatient setting can have long‐term impacts in regard to the cost savings associated with healthcare utilization in the United States. This is increasingly acknowledged by public health and governmental agencies as food insecurity is now a required screening component in the inpatient setting per the Centers for Medicare and Medicaid Services (CMS).^37^ Before this mandate, which was initiated in 2024, addressing food insecurity was recognized, implemented and continues to be refined within this LTACH.

The need for robust screening measures extends beyond the US healthcare system. Food insecurity profoundly affects low‐to‐middle income countries (LMIC), which are classified based on their per capita gross national income.^38^, ^39^ A literature review by Bloem and Farris highlights both the importance and effectiveness of instituting social protection programs, such as cash transfers or in‐kind food payments because they play a protective role in offsetting the detrimental effects of food insecurity within LMICs.^40^ However, the level as well as the duration of social and economic support offered by these programs can vary by community and geographical setting (rural versus urban environments).^40^, ^41^ LMICs can significantly benefit from the implementation of scalable and simplistically designed food insecurity screening protocols. The food insecurity screening protocol that has been developed within this LTACH could feasibly serve as a blueprint or provide a framework for forming a healthcare‐based CHNA in areas vulnerable to food insecurity outside of the US. In utilizing brief, validated food accessibility questionnaires that are culturally appropriate, this CHNA provides a practical model that could be adapted and modified into already‐existing initial consult formats to capture those who are food insecure within various healthcare settings. This could include rehabilitation centers or multidisciplinary team run clinics, as examples.

Specific mechanisms to optimize the CHNA food insecurity screening protocol includes extending data collection to capture sociodemographic information related to race/ethnicity, family size, annual income, rural versus urban residence, native language, religious affiliations, and gender identity upon patient consent. This would provide valuable insight to help inform clinicians as to patient‐centered needs surrounding food insecurity. Moreover, it can support or guide the implementation of culturally sensitive screening questions upon developing a validated, inpatient food insecurity screening instrument that medical staff at any level of care can administer. This is key given that food insecurity is such a sensitive and stigmatized issue. An additional aspect to consider is that clinicians and nursing staff are faced with time constraints on a daily basis. A lengthy and extensive screening tool could very well result in user attrition and nonresponse by the patient, once again leading to the food insecurity component of care falling to the wayside. Minimizing the number of screening questions while maintaining an accurate assessment of food insecurity is a critical implemenation consideration. Lastly, as observed by the medical team within the first quarter of implementation, it is imperative that the patient receives nutrition‐based resources as soon as food insecurity is identified. Completing the screening, providing the materials, and communicating concerns with the medical team is a multistep process. Initiating these steps promptly or upon LTACH admission ensures that every food insecure patient is in fact connected with the appropriate materials and resources prior to their scheduled discharge. The importance of timely dissemination of nutrition resources as soon as food insecurity is identified has been acknowledged and demonstrated by this medical team as evidenced by improvements in year‐end averages in the provision of community nutrition resources from 2022 to 2023 (see Figures 1 and 2 for details).

Recognized limitations of the current CHNA protocol includes the modest number of food insecure patients that have been identified annually within this LTACH. This includes the third quarter of 2023 in which there were no identified food insecure patients. Of note, this coincides with a period in which there was staff turnover, or a new LTACH RD was hired and trained within this facility. Additionally, although every patient was asked about food insecurity status during the initial nutrition consult, there were no standardized food insecurity screening questions in place upon piloting this protocol. However, as the CHNA protocol continues to be refined and further developed, it is recognized by this medical team that incorporating standardized questions as part of a validated questionnaire is an appropriate next step.

### Future directions

Future directions of this LTACH‐based food insecurity screening protocol includes matriculating an existing, validated food insecurity questionnaire that can be rapidly administered to the adult patient population. Food insecurity questionnaires that have been utilized within healthcare settings can vary in length as well as target populations.^42^, ^43^, ^44^ For instance, the US Department of Agriculture (USDA) developed a 10‐item US adult food security screening tool which prompts individuals for how often they skipped meals, experienced hunger, or observed unintended weight loss, among other aspects that are associated with symptoms of food insecurity.^43^ However, a more brief, 2‐question, Hunger Vital Sign™ questionnaire, developed by Hager and Quigg ^44^, ^45^ is an ideal, validated tool that can be utilized in the adult population.^46^, ^47^ The questionnaire asks, “In the past 12 months I/we were worried whether our food would run out before we got money to buy more” and “Within the past 12 months the food I/we bought just didn't last and I/we didn't have money to get more.”^44^

The survey allows for levels of responses including, “Often true,” “Sometimes true,” and “Never true.”^45^ Although questionnaires such as this do not provide insight as to the severity of food insecurity or capture social complexities involved with being food insecure, the brevity of the screening tool makes it a more sustainable practice and can effectively trigger more comprehensive consultations from the ancillary team, such as on‐site social workers.

An additional consideration for this protocol is collecting post‐screening feedback metrics by conducting a Plan‐Do‐Study‐Act (PDSA) cycle to track hospital readmission rates of patients identified as food insecure. This can help test the effectiveness of the protocol and whether it is impacting medical care utilization within this healthcare system. Another PDSA cycle consideration is to establish whether the food insecure patients who were provided the CHNA nutrition‐related resources during their LTACH admission actually utilized and benefited from them post‐discharge. It would be fascinating to explore if there are components of the nutrition‐resource packet that patients are more likely to utilize as this could help inform clinicians as to which elements of the nutrition‐resource packet to place focus on when preparing the patient for hospital discharge.

Another point of discussion in regard to future directions includes coordinating or partnering with local food banks to offer patients nutrient‐dense, nonperishable food items as an additional component of the CHNA nutrition‐resource packet. The tangible elements provided in the nutrition‐resource packet, such as ONS samples and coupons, are highly valued by food insecure patients. Expanding on these resources to include physical recommended food items could serve as a more direct and practical approach to improving outcomes post‐discharge as it explicitly connects the patient with nutritional resources in real time. This strategy has been successfully implemented within other healthcare institutions. For instance, a tertiary hospital system in western New York partnered with a local food bank to create a hospital‐based food pantry as part of their integrated food insecurity protocol.^48^ This is an ideal future outcome which models the potential that hospital‐based food insecurity protocols can have when they gain collaborative support and traction within the community.

## CONCLUSION

In conclusion, food insecurity is an SDoH that is inextricably associated with negative health outcomes and increased healthcare utilization. Within this high acuity, long‐term acute care facility, we were able to implement a food insecurity screening protocol that is now part of the initial nutrition consultation for every newly admitted LTACH patient. This is critical given that these are medically complex and nutritionally at‐risk patients in which optimizing nutrition status is an integral component of their care. The implementation of this protocol sets the initial groundwork that is helping to guide the development of a more effective and scalable food insecurity‐based screening protocol for hospitalized patients. As a way to enhance or improve upon the current screening protocol, ideal next steps include incorporating a brief and validated food insecurity questionnaire within the initial nutrition consultation as well as collect metrics that can inform us of protocol effectiveness.

## AUTHOR CONTRIBUTIONS

Tina Gross and Martha Samsel equally contributed the concept and design of the quality improvement project; Christopher Stimson contributed to data acquisition and editing and drafting of the manuscript for this quality improvement project; Molly MacDonald contributed to the data acquisition, data analysis, and drafting of the manuscript; and all authors have read and approved the final manuscript.

## CONFLICT OF INTEREST STATEMENT

None declared.
